# Evaluating Large Language Models in Ophthalmology: Systematic Review

**DOI:** 10.2196/76947

**Published:** 2025-10-27

**Authors:** Zili Zhang, Haiyang Zhang, Zhe Pan, Zhangqian Bi, Yao Wan, Xuefei Song, Xianqun Fan

**Affiliations:** 1 State Key Laboratory of Eye Health, Department of Ophthalmology Shanghai Ninth People’s Hospital Shanghai Jiao Tong University School of Medicine Shanghai China; 2 School of Computer Science and Technology Huazhong University of Science and Technology Wuhan China

**Keywords:** ophthalmology, large language model, systematic review, meta-analysis, clinical evaluation, artificial intelligence

## Abstract

**Background:**

Large language models (LLMs) have the potential to revolutionize ophthalmic care, but their evaluation practice remains fragmented. A systematic assessment is crucial to identify gaps and guide future evaluation practices and clinical integration.

**Objective:**

This study aims to map the current landscape of LLM evaluations in ophthalmology and explore whether performance synthesis is feasible for a common task.

**Methods:**

A comprehensive search of PubMed, Web of Science, Embase, and IEEE Xplore was conducted up to November 17, 2024 (no language limits). Eligible publications quantitatively assessed an existing or modified LLM on ophthalmology-related tasks. Studies without full-text availability or those focusing solely on vision-only models were excluded. Two reviewers screened studies and extracted data across 6 dimensions (evaluated LLM, data modality, ophthalmic subspecialty, medical task, evaluation dimension, and clinical alignment), and disagreements were resolved by a third reviewer. Descriptive statistics were analyzed and visualized using Python (with *NumPy*, *Pandas*, *SciPy*, and *Matplotlib* libraries). The Fisher exact test compared open- versus closed-source models. An exploratory random-effects meta-analysis (logit transformation; DerSimonian-Laird τ^2^) was performed for the diagnosis-making task; heterogeneity was reported with *I*^2^ and subgrouped by model, modality, and subspecialty.

**Results:**

Of the 817 identified records, 187 studies met the inclusion criteria. Closed-source LLMs dominated: 170 for ChatGPT, 58 for Gemini, and 32 for Copilot. Open-source LLMs appeared in only 25 (13.4%) of studies overall, but they appeared in 17 (77.3%) of evaluation-after-development studies, versus 8 (4.8%) pure-evaluation studies (*P*<1×10^-5^). Evaluations were chiefly text-only (n=168); image-text tasks, despite the centrality of imaging, were used in 19 studies. Subspecialty coverage was skewed toward comprehensive ophthalmology (n=72), retina and vitreous (n=32), and glaucoma (n=20). Refractive surgery, ocular pathology and oncology, and ophthalmic pharmacology each appeared in 3 or fewer studies. Medical query (n=86), standardized examination (n=41), and diagnosis making (n=29) emerged as the 3 predominant tasks, while research assistance (n=5), patient triaging (n=3), and disease prediction (n=3) received less attention. Accuracy was reported in most studies (n=176), whereas calibration and uncertainty were almost absent (n=5). Real-world patient data (n=45), human performance comparison (n=63), non‑English testing (n=24), and real-world deployment (n=4) were relatively absent. Exploratory meta-analysis pooled 28 diagnostic evaluations from 17 studies: overall accuracy was 0.594 (95% CI 0.488-0.692) with extreme heterogeneity (*I*^2^=94.5%). Subgroups remained heterogeneous (*I*^2^>80%), and findings were inconsistent (eg, pooled GPT-3.5>GPT-4).

**Conclusions:**

Evidence on LLM evaluations in ophthalmology is extensive but heterogeneous. Most studies have tested a few closed-source LLMs on text-based questions, leaving open-source systems, multimodal tasks, non-English contexts, and real-world deployment underexamined. High methodological variability precludes meaningful performance aggregation, as illustrated by the heterogeneous meta-analysis. Standardized, multimodal benchmarks and phased clinical validation pipelines are urgently needed before LLMs can be safely integrated into eye care workflows.

## Introduction

### Background

Driven by rapid advancements in natural language processing technology powered by artificial intelligence (AI), large language models (LLMs), such as ChatGPT (OpenAI), are revolutionizing health care. LLMs, trained on diverse, high-quality datasets, encode extensive knowledge, and generate humanlike responses [1], showing promise in disease prevention, diagnosis, treatment, caregiving, and education [2].

Ophthalmic diseases significantly impact global health [3,4], yet a shortage of ophthalmologists, exacerbated by aging populations, has widened the gap between health care demand and supply, particularly in low- and middle-income countries [5]. LLMs are seen as a potential solution to this resource shortage [6], with studies demonstrating their capabilities in ophthalmology. For instance, Antaki et al [7] found that ChatGPT with GPT-4 [8] achieved an accuracy of more than 70% on simulated ophthalmology board-style exams, outperforming historical human performance. Similarly, Bernstein et al [9] demonstrated that LLMs could appropriately respond to patients’ eye health concerns, with response quality comparable to that of ophthalmologists.

However, LLMs are not always reliable [10]; they may generate convincing yet factually incorrect responses [9], posing risks to patient care. Thus, systematic evaluation of LLMs is essential before their clinical integration [6]. While numerous studies have assessed LLMs in ophthalmology [11-13], a comprehensive statistical analysis and synthesis of these evaluation practices is lacking. This gap hinders the standardization of future model evaluations, thereby postponing clinical deployment.

### Aim and Scope of This Review

This study aims to systematically map the current landscape of LLM evaluations in ophthalmology. To achieve this, we first summarized existing LLM evaluation practices in ophthalmology across 6 dimensions: evaluated LLM, data modality, ophthalmic subspecialty, medical task, evaluation dimension, and clinical alignment. Moreover, we performed an additional meta-analysis of diagnostic performance evaluation, providing further evidence for the heterogeneity and fragmentation observed in current evaluation studies. By identifying gaps in current practices, we propose a standardized framework to guide future research and clinical implementation, ensuring the safe and effective integration of LLMs into ophthalmic care.

## Methods

### Design and Registration

A systematic review was conducted in accordance with the relevant sections of the PRISMA (Preferred Reporting Items for Systematic Reviews and Meta-Analyses) reporting guidelines [14], and the completed PRISMA checklist is provided in Multimedia Appendix 1.

### Ethical Considerations

Ethics approval was not required for this study because it did not involve the recruitment of patients.

### Search Strategy

Peer-reviewed studies and preprints were retrieved on November 17, 2024, from 4 databases, including PubMed, Web of Science, Embase, and IEEE Xplore. The key search strategies were based on a combination of 2 themes: LLM and ophthalmology, and synonyms or related terms of these 2 themes were also included in the search strategy to ensure a comprehensive coverage of related publications. The complete search strategies in 4 databases are listed in Multimedia Appendix 2 [15-33].

### Study Selection

All records were screened in 3 sequential phases by 2 independent reviewers (ZZ and ZP). First, an automated title-matching script removed exact duplicates before any screening. Second, during title and abstract screening, reviewers applied the inclusion and exclusion criteria (Textbox 1) and manually discarded near-duplicate records (eg, preprint version of a published study). Third, full texts were assessed against the same criteria, and any additional duplicates were removed when the full-text content was found to be substantially identical despite different titles or abstracts. Disagreements were resolved through discussion, with a third reviewer (HZ) acting as arbiter when necessary.

**Inclusion and exclusion criteria for the systematic review of quantitative large language model (LLM) evaluations in ophthalmology.**

**Inclusion criteria**

- Topic: ophthalmology-related tasks, questions, cases, or images
- Model type: original or modified LLMs (fine-tuned, retrieval-augmented generation enhanced [34], or pipeline integrated)
- Evaluation: 1 or more quantitative metrics (eg, accuracy, Likert scale, and readability score)
- Publication: peer-reviewed studies and preprints; any language

**Exclusion criteria**

- Topic: nonophthalmic content; no artificial intelligence involved
- Model type: traditional deep learning models, such as a convolutional neural network; vision-only generation models, such as DALL·E
- Evaluation: purely qualitative commentaries
- Publication: full text unavailable; preprint version of published study

In line with recent AI-focused reviews [35], we used domains that are more appropriate for AI model evaluations rather than the traditional patient-centered population, intervention, comparison, outcome, and study design headings.

### Data Extraction and Analysis Framework

#### Overview

A wide range of data was extracted and summarized from included publications, mainly across 6 aspects: evaluated LLM, data modality, ophthalmic subspecialty, medical task, evaluation dimension, and clinical alignment. The data extraction was conducted manually by 2 independent reviewers (ZZ and ZP), and a short Python (Python Software Foundation) script was then used to compare the 2 extraction sheets and generate a list of discrepant entries for joint discussion and consensus. Data extraction outcomes are detailed in Multimedia Appendix 3 [2,7,9,11-13,15,16,36-197].

#### Evaluated LLM

We aggregated LLMs included in each study by model series (eg, grouping GPT-3.5-Turbo, GPT-4, and GPT-4o under “ChatGPT Series”), and we also consolidated models with shared technical lineages (eg, categorizing Pathways Language Model [198], Google Bard, and Gemini [199] under “Gemini Series”). Such categorization ensures consistent tracking of research attention despite frequent model updates. Furthermore, we classified each model as open-source LLM (eg, Large Language Model Meta AI [LLaMA] [200] and DeepSeek [201]) or closed-source LLM (eg, ChatGPT and Gemini) based on public availability of weights and code.

#### Data Modality

We categorized the evaluation modalities into 2 types: image-text and text-only. Studies involving any image-related questions were classified under the image-text category.

#### Ophthalmic Subspecialty

A comprehensive classification framework comprising 12 subspecialties and an additional category, “comprehensive ophthalmology,” was established based on multiple authoritative sources, including the American Academy of Ophthalmology, the Royal College of Ophthalmologists, and relevant studies [202,203]. Studies were classified as “comprehensive ophthalmology” if they either did not target a single subspecialty or covered 3 or more distinct subspecialties (eg, cataract, glaucoma, and retina) without a predominant focus on any single domain.

#### Medical Task

Medical tasks across the included studies were classified into 9 distinct categories based on their objectives, target populations, and methodological approaches. This classification framework integrates insights from previous research [204,205], with empirical patterns observed in our literature corpus (refer to definitions in Table S1 in Multimedia Appendix 2).

#### Evaluation Dimension

We adopted the 7D framework proposed by Bedi et al [204] for testing LLMs in health care applications:

1. Accuracy—concordance between the model’s output and a gold-standard reference
2. Comprehensiveness—the extent to which the output addresses all clinically relevant aspects of the prompt
3. Factuality—alignment with perceived consensus and correctness of any cited sources, such as publications and clinical guidelines
4. Robustness—stability of performance under input perturbations, such as typos and paraphrasing, or reproducibility of the answer under repeated identical queries
5. Fairness, bias, and toxicity—absence of harmful, discriminatory, or toxic content toward both majority and ethnic minority groups
6. Deployment metrics—practical considerations, such as latency, computational cost, and memory footprint
7. Calibration and uncertainty—alignment between predicted confidence and actual correctness

Furthermore, “readability and usability” was added as the eighth category to capture an evaluation item that appeared repeatedly in included studies but was not covered earlier. It reflects the linguistic clarity and clinical usability of model outputs for patients or clinicians. This dimension was often assessed with two complementary approaches: (1) objective formulas, most commonly the Simple Measure of Gobbledygook, Flesch-Kincaid Grade Level, and Flesch-Kincaid Reading Ease and (2) subjective instruments, such as the Patient Education Materials Assessment Tool and Likert scale ratings completed by clinicians or patients. Any study using 1 or more of these measures (or equivalent methods) was classified under this dimension.

#### Clinical Alignment

Four dimensions assessed clinical alignment. The “language” dimension recorded the languages used in the evaluation to calculate the proportion of studies that included non-English assessments. The “real patient data included” dimension captured whether studies used real patient data, such as clinical information, examination results, or ophthalmic imaging. The “compared with human performance” dimension indicated whether studies compared the performance of LLMs with that of ophthalmologists. Human responses could either be generated specifically for the study or sourced from existing online content. Finally, the “real-world clinical assessment” dimension evaluated whether studies involved deployment, application, and assessment of LLMs in real clinical settings rather than limiting testing to virtual environments.

A study may contain multiple evaluated LLMs, ophthalmic subspecialties, medical tasks, evaluation dimensions, and languages. Multiple results were separated by a slash during data extraction, and each was included in the statistical results; therefore, the sum of their percentages exceeded 100%. “Uncertain” was used in data extraction when the result could not be determined from the full text of the paper.

### Exploratory Meta-Analysis

We performed an exploratory meta-analysis limited to the diagnosis task, the only domain with relatively uniform methodology. Eligible studies were screened using 2 criteria: reporting an exact “correct or incorrect” proportion on open-ended diagnostic questions and avoiding multiple-choice or top-N scoring formats. The proportion of correct diagnoses was the primary effect size. Parallel model arms within a study were treated as independent evaluations. Data, including model, case count, modality, subspecialty, and accuracy, were extracted through dual-independent review with third-reviewer adjudication. Random-effects pooling (logit transformation; DerSimonian-Laird τ^2^) and inverse-variance weighting were applied in Python (version 3.8.19) using NumPy (version 1.24.3) and SciPy (version 1.10.1) libraries, and heterogeneity was quantified with *I*^2^. Subgroup analyses by LLM, modality, and subspecialty were performed to explore sources of heterogeneity.

### Statistical Analysis and Visualization

Bar plots and stacked bar plots were used to visualize the distribution of relevant studies across different categories, and forest plots were generated for the exploratory meta-analysis. A 2-sided Fisher exact test compared the prevalence of open-source LLMs in “evaluation-after-development” studies versus “pure-evaluation” studies. Data statistics and visualization were conducted in Python (version 3.8.19) using NumPy (version 1.24.3), Pandas (version 2.0.3, SciPy (version 1.10.1), and Matplotlib (version 3.7.3) libraries.

## Results

### Overview

**Figure 1:**
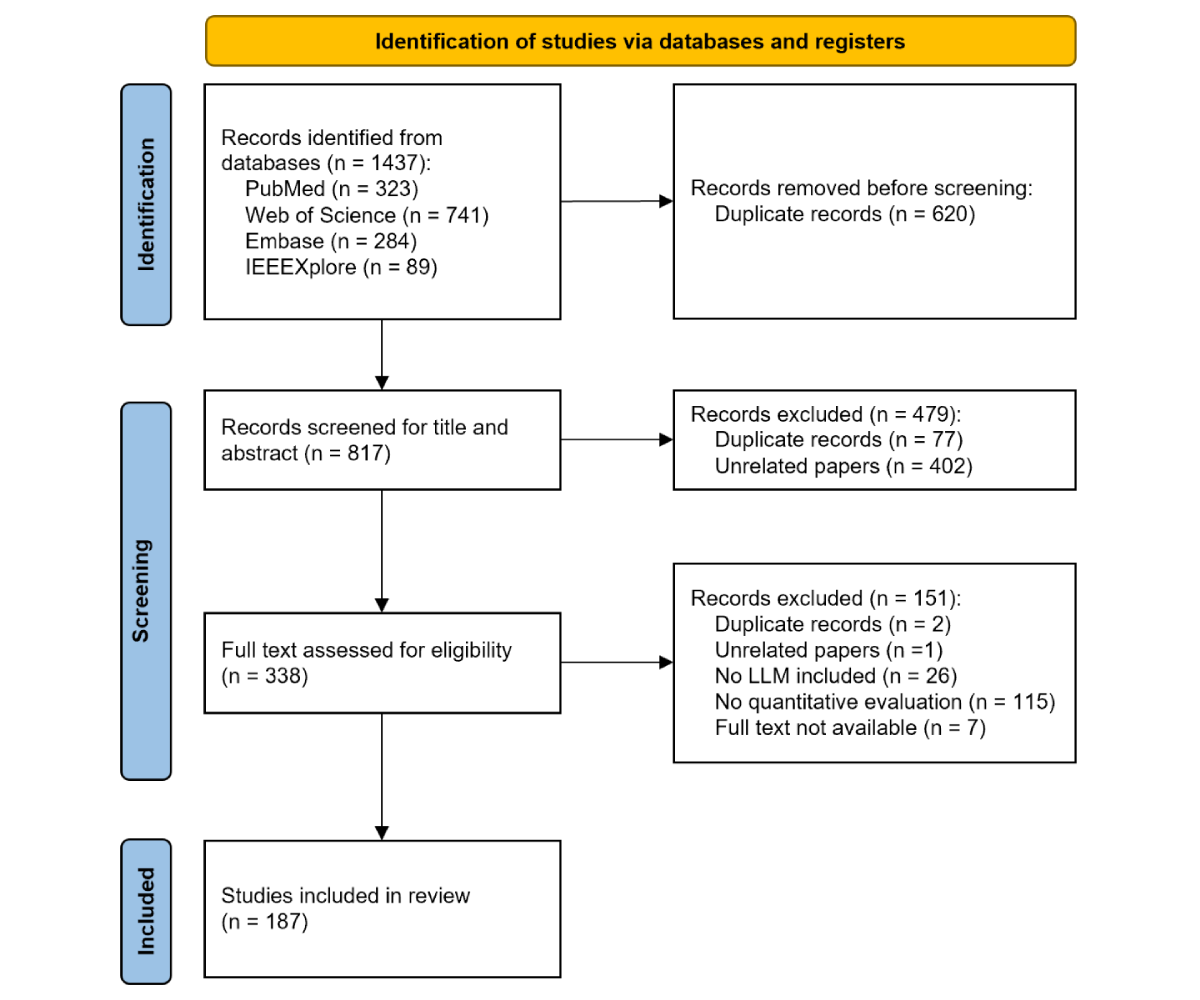
PRISMA flow diagram of the article screening and identification process. The figure depicts identification, screening, eligibility, and inclusion of 187 peer-reviewed or preprint studies that quantitatively evaluated large language model (LLM) performance for ophthalmic tasks. Searches were conducted in PubMed, Web of Science, Embase, and IEEE Xplore on  November 17,  2024, with no language limits. Reasons for exclusion at each stage are shown.

A total of 817 unique records were identified through our systematic search. After removing duplicates and unrelated papers based on title and abstract, 338 (41.4%) studies remained for full-text screening. Ultimately, a total of 187 (22.9%) studies met the study selection criteria and were included in the analysis (Figure 1). Key features of the included studies are described in Table 1.

**Table 1:** Key features of included publications in this study (N=187).

Category and subtype			Studies, n (%)
**Publication venue**			
	Peer-reviewed journal	177 (94.7)	
	Preprint platform (eg, arXiv and medRxiv)	9 (4.8)	
	Conference	1 (0.5)	
**Publication year**			
	2024	143 (76.5)	
	2023	44 (23.5)	
**Evaluation type**			
	Pure evaluation^a^	165 (88.2)	
	Evaluation after development^b^	22 (11.8)	

^a^Evaluating base large language models without any architectural modification (eg, ChatGPT via its web interface).

^b^Evaluating large language models that were fine-tuned, retrieval-augmented generation enhanced, or integrated into new pipelines within the same study.

### Evaluated LLM

Closed-source LLMs overwhelmingly dominated the included studies in this review. In total, 169 (90.4%) of the 187 studies evaluated 1 to 3 models, and more than half (n=100, 53.5%) compared multiple LLMs in parallel (Figure S1 in Multimedia Appendix 2). The 3 most frequently assessed LLM series were all closed-source ones—ChatGPT (n=170, 90.9%), Gemini (n=58, 31%; including Pathways Language Model, Bard, and Gemini), and Copilot (n=32, 17.1%; including Copilot [206] and Bing AI). In contrast, open-source LLMs were only evaluated in 25 (13.4%) studies, for example, LLaMA (n=15, 8%) and ChatGLM [207] (n=6, 3.2%; Table 2).

**Table 2:** Study characteristics for ophthalmology large language model (LLM) evaluations—evaluated LLM, data modality, and ophthalmic subspecialty (N=187).

Category and subtype		Studies^a^, n (%)
**LLM series (top 5 most frequently evaluated)**		
	ChatGPT	170 (90.9)
	Gemini	58 (31)
	Copilot	32 (17.1)
	LLaMA^b^	15 (8)
	ChatGLM	6 (3.2)
**Data modality**		
	Text-only	168 (89.8)
	Image-text	19 (10.2)
**Ophthalmic subspecialty**		
	Comprehensive ophthalmology	72 (38.5)
	Retina and vitreous	32 (17.1)
	Glaucoma	20 (10.7)
	External disease and cornea	12 (6.4)
	Pediatric ophthalmology and strabismus	11 (5.9)
	Lens and cataract	8 (4.3)
	Oculoplastics and orbit	8 (4.3)
	Uveitis and ocular inflammation	7 (3.7)
	Neuro-ophthalmology	7 (3.7)
	Clinical optics and vision rehabilitation	6 (3.2)
	Refractive surgery	3 (1.6)
	Ophthalmic pharmacology	2 (1.1)
	Ocular pathology and oncology	2 (1.1)

^a^The sum of percentages of evaluated LLM series and ophthalmic subspecialties exceeds 100% because a study may be categorized into more than 1 classification (eg, a study evaluated both ChatGPT and Gemini).

^b^LLaMA: Large Language Model Meta AI.

Notably, open-source models accounted for a substantially larger share of “evaluation-after-development” studies than “pure-evaluation” studies (17/22, 77.3% vs 8/165, 4.8%; *P*<1×10^-5^, Fisher exact test; Figure S2 in Multimedia Appendix 2). Detailed definitions of the 2 study types (Table 1) demonstrate superior domain-specific versatility in ophthalmology.

### Data Modality

Image-based evaluations were uncommon. Only 19 (10.2%) of the 187 studies assessed the ability of LLMs to process images and text in ophthalmology, whereas most (n=168, 89.8%) focused solely on text-only performance (Table 2). The ophthalmic images involved in these multimodality evaluations include, but are not limited to, slit lamp images, fundus photography of the posterior pole, optical coherence tomography, and ophthalmic ultrasonography. Other modalities such as voice, video, or documents were not included in any of the studies.

### Ophthalmic Subspecialty

Evaluations clustered in comprehensive ophthalmology (72/187, 38.5%), leaving many subspecialties scarcely explored. Retina and vitreous (32/187, 17.1%) and glaucoma (20/187, 10.7%) followed at a distance, whereas ocular pathology and oncology, refractive surgery, and ophthalmic pharmacology appeared in no more than 3 studies each (Table 2).

### Medical Task

**Figure 2:**
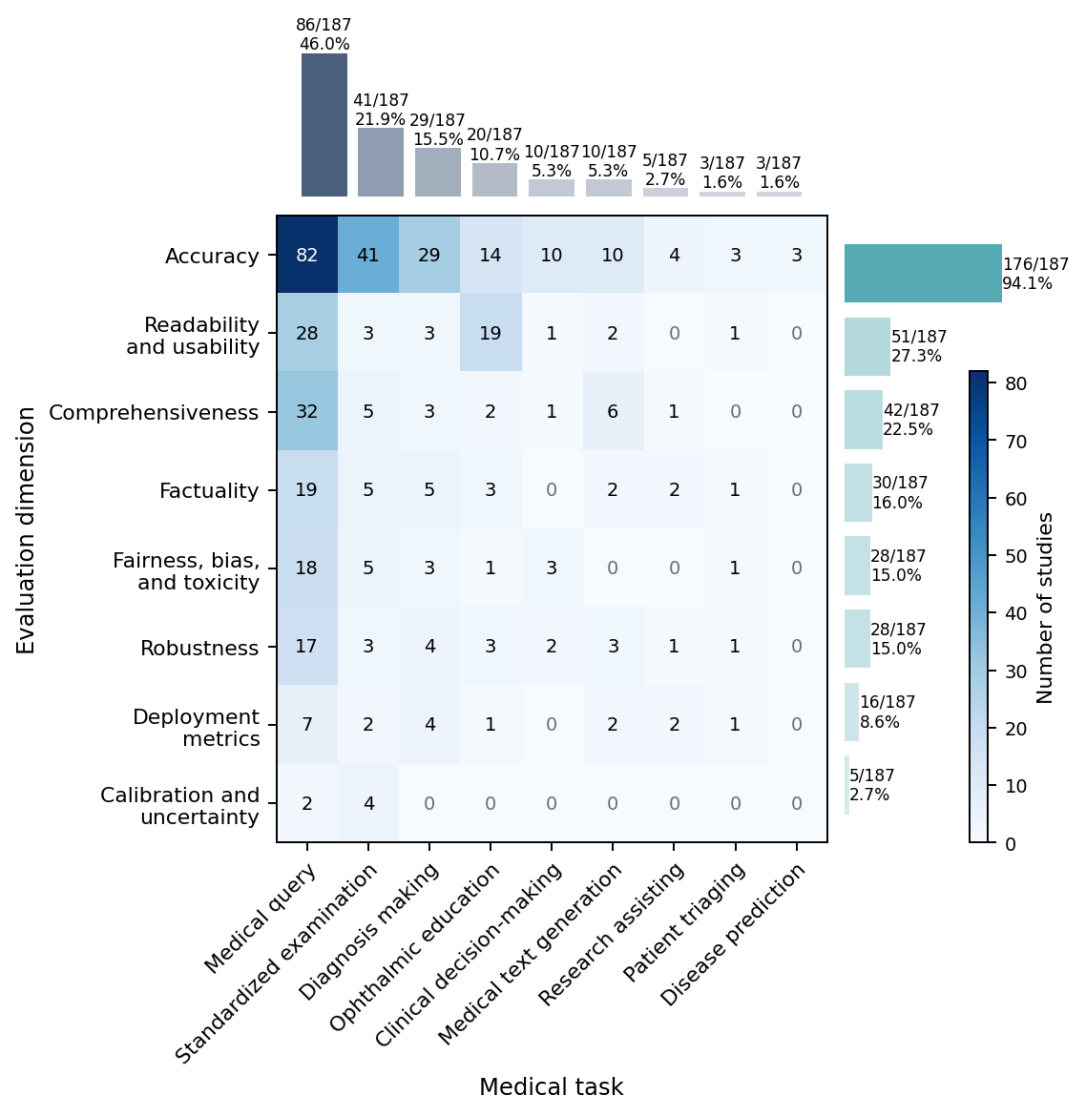
Heat map of evaluation dimension by medical tasks across 187 ophthalmology large language model studies.

Medical task coverage was unevenly distributed. Most studies concentrated on medical query (86/187, 46.0%), standardized examination (41/187, 21.9%), and diagnosis making (29/187, 15.5%) in ophthalmology, and other common tasks explored (>5%) included ophthalmic education, clinical decision-making, and medical text generation. In contrast, research assisting (5/187, 2.7%), patient triaging (3/187, 1.6%), and disease prediction (3/187, 1.6%) received comparatively less attention (Figure 2).

### Evaluation Dimension

Evaluation dimensions were concentrated and unevenly distributed. Among 187 included studies, 118 (63.1%) evaluated 2 or more dimensions, yet only 15 (8%) assessed 4 or more dimensions, demonstrating the comprehensiveness of the existing evaluation (Figure S3 in Multimedia Appendix 2). Accuracy (n=176, 94.1%) was the most prevalent evaluation dimension, whereas calibration and uncertainty (n=5, 2.7%) were the least frequently examined. Notably, specific medical tasks showed distinct preferences for evaluation dimensions. For example, among 20 studies on ophthalmic education, 19 (95.0%) assessed readability and usability (Figure 2).

### Clinical Alignment

**Figure 3:**
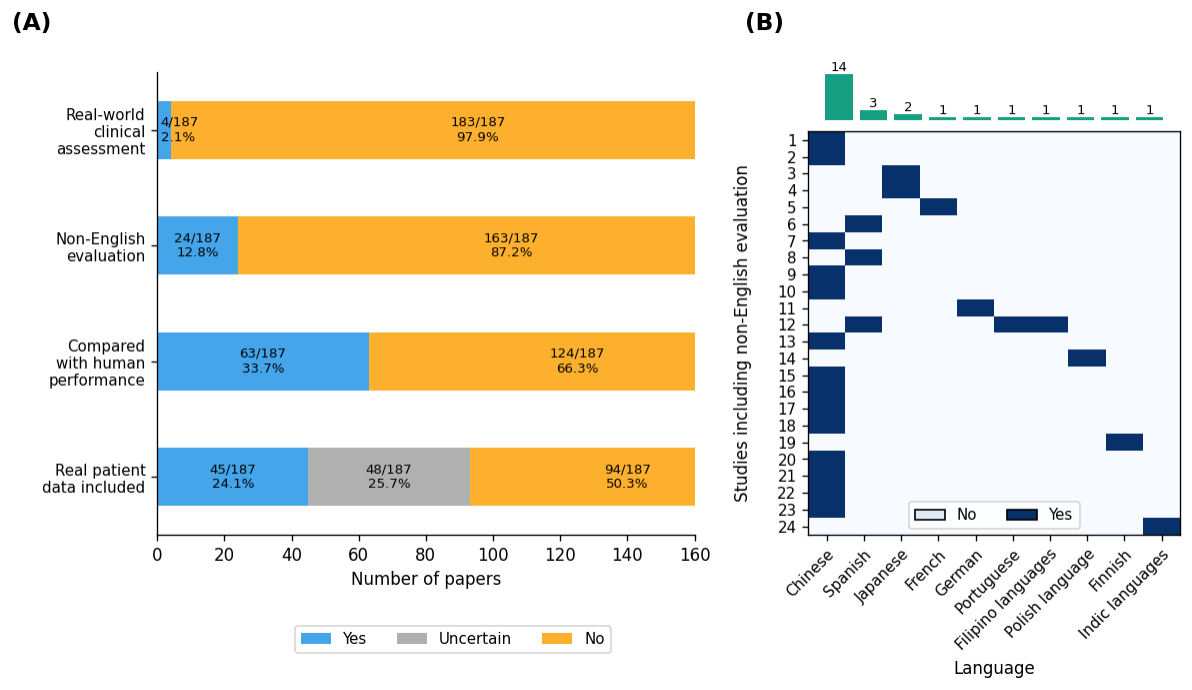
Clinical-alignment landscape of ophthalmology large language model (LLM) studies. (A) Shows the proportion of papers that did or did not involve real patient data, comparison with human performance, non-English evaluation, and in-clinic deployment in evaluation practice. (B) Among the 24 papers that conducted non-English evaluation, the heat map shows which of 10 language groups were tested (rows=studies), with the top bar summarizing language frequency.

The use of real patient data was limited and often undocumented. As Figure 3A shows, of the 187 studies, 45 (24.1%) tested LLMs with real patient data, including clinical information, examination results, and imaging; 48 (25.7%) studies did not provide enough methodological detail to ascertain the data source; the remaining 94 (50.3%) studies used purely non–case-based ophthalmic problems or virtual patient data.

Comparisons between LLMs and humans were common but not universal. As Figure 3A shows, of the 187 studies, 63 (33.7%) compared LLM outputs with the performance of ophthalmologists at various training levels, providing a reference point for potential clinical integration.

Multilingual evaluations were scarce. As Figure 3A shows, only 24 (12.8%) of the 187 studies tested LLMs in non-English contexts. As Figure 3B shows, of these 24 studies, Chinese (n=14, 58.3%), Spanish (n=3, 12.5%), and Japanese (n=2, 8.3%) languages dominated these assessments; the remaining 7 languages (eg, French, German, and Finnish) appeared just once each.

Evaluations under real-world deployment were exceedingly rare. As Figure 3A shows, only 2.1% (4/187) of the studies evaluated LLMs in real ophthalmic clinical settings, while the remaining (183/187, 97.9%) studies were all conducted based on simulated clinical scenarios.

### Exploratory Meta-Analysis of Diagnosis-Making Studies

In total, 17 (58.6%) of the 29 diagnosis-making studies met our stricter inclusion criteria, providing 28 independent LLM evaluations of open-ended diagnostic accuracy (Figure S4 in Multimedia Appendix 2). Data extraction outcomes are detailed in Table S2 in Multimedia Appendix 2. Random-effects pooling yielded an overall correct-diagnosis proportion of 0.594 (95% CI 0.488-0.692) with extreme heterogeneity (*I*^2^=94.5%). Forest plots are provided in Figure S5 in Multimedia Appendix 2. Subgroup pooling did not resolve this variability: *I*^2^ values remained greater than 80% for most models, modalities, and subspecialty strata (Table 3).

MOPH achieved the highest accuracy for diagnosis, though this estimate was based on a single evaluation. Counterintuitively, GPT-4 (pooled accuracy=0.559) underperformed compared to GPT-3.5 (pooled accuracy=0.649). Across modalities, the pooled accuracy for image-text tasks (0.407) was lower than for text-only tasks (0.619). Five subspecialties and “comprehensive ophthalmology” were included in the subspecialty analysis; pooled estimates ranged from 0.483 for retina and vitreous to 0.731 for external disease and cornea, all with wide confidence intervals.

**Table 3:** Pooled overall and subgroup diagnostic accuracy.

			Pooled accuracy (95% CI)		*I* ^2^		Evaluations, n^a^
Overall			0.594 (0.488-0.692)		*94.5* ^b^		28
**Subgroups: model**							
	MOPH^c^	0.811 (0.747-0.862)		—^d^		1	
	GPT-3.5	0.649 (0.503-0.772)		*90.6*		12	
	GPT-4	0.559 (0.402-0.704)		*93.2*		9	
	Bing Copilot	0.526 (0.275-0.765)		24.1		2	
	Bard	0.438 (0.333-0.547)		—		1	
	Gemini	0.421 (0.226-0.644)		—		2	
	Glass 1.0	0.333 (0.084-0.732)		—		1	
**Subgroup: modality**							
	Text-only	0.619 (0.522-0.708)		*91.9*		25	
	Image-text	0.407 (0.123-0.770)		*96.9*		3	
**Subgroup: subspecialty**							
	External disease and cornea	0.731 (0.427-0.909)		66		2	
	Glaucoma	0.727 (0.414-0.910)		—		1	
	Comprehensive ophthalmology	0.601 (0.503-0.692)		*86.2*		12	
	Uveitis and ocular inflammation	0.589 (0.468-0.701)		—		5	
	Neuro-ophthalmology	0.547 (0.369-0.715)		51.9		5	
	Retina and vitreous	0.483 (0.087-0.901)		*99.4*		3	

^a^Represents the number of evaluations included in the analysis and not the number of studies.

^b^Italics indicate *I*^2^ values of more than 80%, indicating substantial heterogeneity.

^c^MOPH: LLM of ophthalmology.

^d^For a single line (k=1), *I*^2^ cannot be calculated.

## Discussion

### Principal Findings

Our synthesis of 187 studies shows a skewed evaluation landscape; certain aspects, such as question-and-answer tasks, comprehensive ophthalmology, and accuracy, dominate, whereas image-based testing, non-English settings, and low-incidence subspecialties (eg, ocular oncology [208]) are rarely explored. Commonly studied areas share 3 traits: broad task generality, easily accessible test data, and high disease prevalence [209-211]. Neglected topics often lack public datasets and have smaller specialist communities [212,213].

Few (19/187, 10.2%) studies included images despite imaging being central to eye care [214]; technical barriers (clinically usable multimodal LLMs only began to emerge in 2023 and are still maturing [8,199,215,216]), privacy concerns, and data-sharing problems all contribute to this low inclusion rate [217]. Open-source LLMs were underrepresented despite their several irreplaceable advantages over closed-source ones in clinical settings (Table 4), yet they appeared much more frequently after new model development, confirming their suitability for customization for specific ophthalmic tasks (eg, fundus image analysis [36-38]). Unlike pure evaluation studies that mainly relied on manual assessment with Likert scales [2,15,39], these postdevelopment evaluations used automated metrics, such as Bilingual Evaluation Understudy [218] and *F*_1_-score, which are standard metrics in AI and natural language processing domains [40,41], enabling rapid testing after LLM customization.

Profound heterogeneity was observed in current evaluation practice and evidence, driven by 3 factors. The first factor was metric heterogeneity. Even within our unified 8D framework, measures under the same heading were not directly comparable; for example, “accuracy” could be reported as an exact-correct ratio, area under the curve, *F*_1_-score, or a Likert scale score with varying anchors. The second factor was data heterogeneity. Assessment sets differed widely; in the “medical query” task, questions spanned multiple difficulty levels, originated from diverse sources, and covered numerous ophthalmic subspecialties. The third factor was model heterogeneity. LLMs are updated frequently (eg, GPT-4o 2024-05-13, 2024-08-06, 2024-11-20), yet most papers note only the major version, obscuring performance shifts between iterations.

**Table 4:** Comparison of characteristics between open-source large language models (LLMs) and closed-source LLMs.

Key aspect	Open-source LLMs	Closed-source LLMs
Examples	LLaMA^a^ and DeepSeek	ChatGPT and Gemini
Costs	Free of charge	Charge occasionally, depending on use
Performance	Remains a performance gap compared to closed-source models, though significant progress has been made in narrowing it [219]	State-of-the-art performance
Data privacy	Enables on-premises data processing, keeping sensitive information internal	Data are transmitted through cloud APIs^b^, posing risks of third-party exposure
Accessibility	Available to the public, allowing free downloading, deployment, and use	Only available for use without providing access to their underlying code
Customizability	Tailorable for medical terminology, imaging diagnostics, and hospital workflows [216,220,221]	Limited adaptation to special medical needs (eg, 3D CT^c^ or MRI^d^ scan analysis)
Interactive interface	Typically provides basic interfaces, with advanced features dependent on community-driven tools or custom development efforts	Well-developed and feature-rich interfaces

^a^LLaMA: Large Language Model Meta AI.

^b^API: application programming interface.

^c^CT: computed tomography.

^d^MRI: magnetic resonance imaging.

To probe whether pooling was feasible despite this variability, we focused on the methodologically most consistent task, diagnosis making, as more than half of these studies (17/29, 59%) used open-ended questions with binary correct or incorrect scoring. However, the pooled accuracy of 0.594 still demonstrated extreme heterogeneity (*I*^2^=94.5%), indicating that this aggregate statistic failed to represent almost any specific clinical context. Subgroup pooling offered little relief. Some findings were merely self-evident (image-text tasks were harder than text-only tasks), others were contradictory (GPT-3.5 pooled accuracy was higher than GPT-4, probably because 1 study of 422 unusually difficult cases tested GPT-4 alone [16]), and most confidence intervals remained wide. Finally, continuous, undocumented model updates, such as minor ChatGPT and Gemini releases, occur every few months, making any version-based synthesis obsolete almost as soon as it is published. Taken together, these observations confirm that high methodological heterogeneity, small task-specific samples, and model drift currently preclude meta-analytic results from informing bedside use or system refinement. Therefore, rigorous, standardized, and multidimensional benchmarks must precede any performance aggregation if future syntheses are to provide actionable clinical guidance in ophthalmology.

### Comparison to Prior Work

Previous reviews focused on LLM applications in ophthalmology, but to the best of our knowledge, none systematically analyzed evaluation practice itself [6,222-224]. While cross-disciplinary surveys in medicine [204,205] flagged generic gaps in LLM evaluation, our study is the first to quantify them within ophthalmology.

We deepened the analysis by anchoring it in ophthalmology’s distinctive context. Firstly, we gave special weight to multimodal (image-text) evaluation because diagnosis hinges on imaging. Moreover, we paid special attention to non-English evaluation to assess global applicability because eye care resources are unevenly distributed across language regions [225]. Finally, we divided the field into highly granular subspecialties (eg, retina, glaucoma, and ocular oncology), which revealed differences in the degree of attention given to different subspecialties. To capture cutting-edge technical advances, we also expanded our search strategy to include the IEEE Xplore augmentation database, which yielded more than a 5-fold increase in included ophthalmology literature (from 36 to 187 studies) compared to previous reviews [204].

### Road to Real-World Deployment and Evaluation

#### Overview

Only 4 (2.1%) of the 187 ophthalmology studies deployed and evaluated LLMs in real clinical workflows [11,36,42,43], none as a randomized controlled trial (RCT), although RCTs are widely recognized as the gold standard for clinical evidence [226]. We have summarized the obstacles hindering the clinical deployment of LLMs into 10 points, as shown in Table 5. To bridge the LLM implementation-evaluation gap in real-world ophthalmology, we advocate a progressive 3-phase validation road map that keeps pace with rapid LLM iteration while safeguarding patients.

**Table 5:** Ten key obstacles to clinical large language model (LLM) deployment and evaluation.

Obstacle	Explanation	Illustrative example
Domain and modality limitations [227-231]	General-purpose text models often lack specialized medical knowledge or multimodal integration needed for comprehensive care.	A text-only LLM cannot incorporate fundus images, so it misclassifies macular edema severity compared with an ophthalmic multimodal model.
Lack of clinical validation [204,229,230]	Most LLM prototypes have never been tested in prospective, real-world patient care, so their safety and effectiveness remain unproven.	A hospital pilots an LLM for discharge summaries; after limited laboratory tests, it occasionally omits critical drug‐interaction warnings that would be caught in a full clinical trial.
Hallucinations and errors [227-229,231,232]	LLMs can exhibit confidence while generating detailed content that is partially or entirely incorrect.	When asked for glaucoma referral criteria, the model invents a nonexistent “stage 4 angle-closure” classification.
Bias and fairness [227-229,232]	Training data–inherited biases can lead the model to provide systematically different or harmful recommendations for certain groups.	The LLM underrecommends diabetic retinopathy screening intervals for patients in minority groups because those groups were underrepresented in the fine-tuning data.
Data privacy and security [227-232]	Using patient text or images with external AI^a^ services risks breaches of confidentiality and regulatory noncompliance.	Ophthalmologists paste private information into a cloud-hosted chatbot that stores inputs for retraining.
Transparency and explainability [227-230,232]	Clinicians and regulators cannot easily trace how or why the model produced a given answer, limiting trust and auditability.	A physician cannot determine which references the LLM used when it advises against a particular antibiotic, so the advice is ignored.
Regulatory gaps [227,229]	Existing medical device rules do not clearly cover general-purpose, continuously updated LLMs.	An updated model version “drifts” after release; no current pathway obliges the vendor to recertify performance each time weights change.
Legal and liability issues [229,231]	It is unclear who is responsible if an LLM’s output harms a patient or infringes intellectual property rights.	After following chatbot-generated postoperative instructions, a patient is hospitalized, raising questions of clinician versus vendor liability.
Clinician and patient trust and adoption [232]	Users hesitate to rely on opaque tools that may err, so adoption stalls without clear evidence and oversight.	Nurses stop using an LLM triage assistant after noticing several inappropriate urgency ratings.
Human-AI interaction and usability [228,229]	Poor prompt design, ambiguous outputs, or workflow friction can negate any theoretical performance advantage.	A radiologist must craft complex prompts to extract a usable structured report, making the tool slower than manual dictation.

^a^AI: artificial intelligence.

#### Phase 1: Technical Benchmarking

In the first phase, one should start with an open, version-controlled benchmark of high quality, which could be described as an “OphthoQA” analogue to widely accepted medical LLM benchmarks, such as MedQA [233] or PubMedQA [234], but centered on multimodal ophthalmic tasks. The suite should mix board-style questions, deidentified ophthalmic images, and short clinical vignettes, all autoscored and posted to a public leaderboard so each new LLM update can be retested within hours, mitigating the reproducibility crisis that arises when LLMs change their answers over time [235], whereas traditional evaluation cycles take months to refresh the data.

#### Phase 2: Retrospective Validation

In the second phase, one should use large, deidentified electronic health record and image archives to compare LLM outputs (diagnoses, summaries, and triage labels) with gold-standard outcomes or expert panels. The assessment conducted by Sarvari et al [236] of LLMs on the MIMIC-IV [237] dataset exemplifies this approach, and ophthalmology already has smaller resources, such as OCTCases for real-world imaging tests [44]. On-premise inference with open-source LLMs allows each institution to assess its own patient data without private information leaving the firewall.

#### Phase 3: Prospective Trials

In the third phase, one should progressively embed the LLM in clinical workflow as follows: (1) silent-mode logging visible only to investigators; (2) pilot decision-support with mandatory human override; (3) full RCTs measuring diagnostic accuracy, workflow impact, and safety events, with real-time adverse-event monitoring. A recent 2‑arm, open‑label RCT of a fine‑tuned mental health chatbot, featuring continual human review and immediate escalation of unsafe output, demonstrated a viable template [238]. This design offers a valuable reference that future ophthalmology-focused LLM trials can adapt to their specific clinical context. Only after a successful RCT should an LLM be adopted in routine eye care, ensuring maximum benefit and minimum harm to patients.

We hope this road map will provide valuable guidance for developing, evaluating, and refining ophthalmology-specific LLMs, ultimately enhancing ophthalmic clinical workflows and improving patient outcomes.

### Future Directions

We propose 4 potential directions for future research. The first direction is the cocreation of a publicly available benchmark for LLMs in ophthalmology, covering all subspecialties, multiple languages, and image-text tasks. The second direction is the adoption of a standard reporting checklist that requires multidimensional metrics (eg, accuracy, comprehensiveness, and safety) and specifies the exact LLM version. The third direction is continuous evaluation of newly launched LLMs, such as the pioneering reasoning model o1, especially open-source models amenable to ophthalmic fine-tuning (eg, DeepSeek and Qwen [239]). The fourth direction includes clinically driven assessments targeting real-world needs, such as referral triage, postoperative counseling, and low-resource language support, progressing through the 3-phase road map to RCTs.

### Limitations

First, the search window closed on November 17, 2024, so publications released afterward, particularly work on the latest LLMs, were not captured. This was especially important given the rapid iteration of recent LLMs. We partially mitigated this by including preprints and 4 multidisciplinary databases, yet progress after the cutoff may have been underestimated.

Second, categorizing medical tasks and evaluation dimensions required subjective judgment; dual-independent extraction with third-reviewer adjudication reduced but did not eliminate misclassification risk.

Third, although we implemented stringent inclusion criteria, the absence of a formal risk-of-bias (RoB) assessment, reflecting the current lack of validated RoB tools specifically for medical AI studies, necessitated equal weighting of all included studies irrespective of methodological rigor. This limitation underscores the need for developing AI-specific RoB frameworks that enable future systematic reviews to appropriately weight evidence by study quality.

Fourth, extreme heterogeneity across studies precluded a definitive meta-analysis, highlighting the need for standardized evaluation protocols and datasets.

### Conclusions

This systematic review mapped 187 ophthalmology-focused LLM evaluations and found a landscape that was extensive yet uneven. Research attention was concentrated on a few closed-source models, predominantly text-based tasks, and a limited set of subspecialties, medical tasks, and evaluation dimensions. Open-source LLMs, multimodal assessments, non-English testing, and real-world clinical studies were scarce. Fragmented methods yield heterogeneous evidence, impeding confident clinical adoption. These findings emphasize the necessity of standardized evaluation frameworks and highlight the critical gaps that must be closed before LLMs can be integrated into ophthalmic practice.

## Appendix

Multimedia Appendix 1 PRISMA 2020 checklist.

Multimedia Appendix 2 Search strategies and supplementary figure and table.

Multimedia Appendix 3 Data extraction results.

## Abbreviations

AI: artificial intelligence
LLaMA: Large Language Model Meta AI
LLM: large language model
PRISMA: Preferred Reporting Items for Systematic Reviews and Meta-Analyses
RCT: randomized controlled trial
RoB: risk of bias

## Data Availability

All data generated or analyzed during this study are included in this published paper and its multimedia appendix files.
