# Supplementary material for: Evaluating Large Language Models in Ophthalmology: Systematic Review
Source: J Med Internet Res. 2025 Oct 27;27:e76947. doi: 10.2196/76947 (PMC12603593; doi:10.2196/76947)
Supplement: Multimedia Appendix 2 [file jmir_v27i1e76947_app2.docx]

**Supplemental Online Content**

Method S1. Search terms for PubMed, Web of Science, Embase and IEEEXplore as of 11/17/2024.

Table S1. Detailed definitions for the nine “Medical Task” labels.

Table S2. Study‑level input data and effect‑size calculations for the diagnosis‑making task meta‑analysis.

Figure S1. Number of LLMs evaluated per included study.

Figure S2. Proportion of studies evaluating open-source LLMs.

Figure S3. Number of evaluation dimensions per included study.

Figure S4. Study selection flow diagram for the diagnosis task meta-analysis.

Figure S5. Forest plot of pooled diagnostic accuracy for diagnosis-making tasks.

**Method S1. Search terms for PubMed, Web of Science, Embase and IEEEXplore as of 11/17/2024**

**Search terms for PubMed.**

("Large Language Model*"[Title/Abstract] OR "LLM"[Title/Abstract] OR "Chatbot*"[Title/Abstract] OR "ChatGPT"[Title/Abstract] OR "Generative AI"[Title/Abstract]) AND ("ophthalmolog*"[Title/Abstract] OR "Eye"[Title/Abstract] OR "Eye Diseases"[MeSH] OR "Asthenopia"[Title/Abstract] OR "Cogan Syndrome"[Title/Abstract] OR "Conjunctival Neoplasms"[Title/Abstract] OR "Conjunctivitis"[Title/Abstract] OR "Pemphigoid"[Title/Abstract] OR "Benign Mucous Membrane"[Title/Abstract] OR "Pinguecula"[Title/Abstract] OR "Pterygium"[Title/Abstract] OR "Xerophthalmia"[Title/Abstract] OR "Conjunctival Disease*"[Title/Abstract] OR "Hereditary Corneal Dystroph*"[Title/Abstract] OR "Corneal Edema"[Title/Abstract] OR "Corneal Endothelial Cell Loss"[Title/Abstract] OR "Corneal Injuries"[Title/Abstract] OR "Corneal Neovascularization"[Title/Abstract] OR "Corneal Opacity"[Title/Abstract] OR "Corneal Wavefront Aberration"[Title/Abstract] OR "Iridocorneal Endothelial Syndrome"[Title/Abstract] OR "Keratitis"[Title/Abstract] OR "Keratoconus"[Title/Abstract] OR "Limbal Stem Cell Deficiency"[Title/Abstract] OR "Trachoma"[Title/Abstract] OR "Corneal Disease*"[Title/Abstract] OR "Aniridia"[Title/Abstract] OR "Anophthalmos"[Title/Abstract] OR "Blepharophimosis"[Title/Abstract] OR "Choroidal Effusion*"[Title/Abstract] OR "Coloboma"[Title/Abstract] OR "Ectopia Lentis"[Title/Abstract] OR "Familial Exudative Vitreoretinopathies"[Title/Abstract] OR "Fraser Syndrome"[Title/Abstract] OR "Hydrophthalmos"[Title/Abstract] OR "Microphthalmos"[Title/Abstract] OR "Persistent Hyperplastic Primary Vitreous"[Title/Abstract] OR "Retinal Dysplasia"[Title/Abstract] OR "Eye Abnormalit*"[Title/Abstract] OR "Aicardi Syndrome"[Title/Abstract] OR "Albinism"[Title/Abstract] OR "Choroideremia"[Title/Abstract] OR "Cone Dystroph*"[Title/Abstract] OR "Cone-Rod Dystroph*"[Title/Abstract] OR "Duane Retraction Syndrome"[Title/Abstract] OR "Familial Exudative Vitreoretinopath*"[Title/Abstract] OR "Graves Ophthalmopathy"[Title/Abstract] OR "Gyrate Atrophy"[Title/Abstract] OR "Leber Congenital Amaurosis"[Title/Abstract] OR "Hereditary Optic Atroph*"[Title/Abstract] OR "Optic Nerve Hypoplasia"[Title/Abstract] OR "Retinal Degeneration"[Title/Abstract] OR "Retinitis Pigmentosa"[Title/Abstract] OR "Retinoblastoma"[Title/Abstract] OR "Stargardt Disease"[Title/Abstract] OR "Walker-Warburg Syndrome"[Title/Abstract] OR "Weill-Marchesani Syndrome"[Title/Abstract] OR "Hereditary Eye Disease*"[Title/Abstract] OR "Choroid Hemorrhage"[Title/Abstract] OR "Hyphema"[Title/Abstract] OR "Retinal Hemorrhage"[Title/Abstract] OR "Vitreous Hemorrhage"[Title/Abstract] OR "Eye Hemorrhage"[Title/Abstract] OR "Corneal Ulcer"[Title/Abstract] OR "Endophthalmitis"[Title/Abstract] OR "Bacterial Eye Infection*"[Title/Abstract] OR "Fungal Eye Infection*"[Title/Abstract] OR "Parasitic Eye Infection*"[Title/Abstract] OR "Viral Eye Infection*"[Title/Abstract] OR "Eye Infection*"[Title/Abstract] OR "Anterior Capsular Rupture"[Title/Abstract] OR "Corneal Injur*"[Title/Abstract] OR "Posterior Capsular Rupture"[Title/Abstract] OR "Eye Injur*"[Title/Abstract] OR "Eye Pain"[Title/Abstract] OR "Lagophthalmos"[Title/Abstract] OR "Eye Manifestation*"[Title/Abstract] OR "Eyelid Neoplasms"[Title/Abstract] OR "Orbital Neoplasms"[Title/Abstract] OR "Paraneoplastic Syndrome"[Title/Abstract] OR "Retinal Neoplasms"[Title/Abstract] OR "Uveal Neoplasms"[Title/Abstract] OR "Eye Neoplasm*"[Title/Abstract] OR "Blepharitis"[Title/Abstract] OR "Blepharoptosis"[Title/Abstract] OR "Blepharospasm"[Title/Abstract] OR "Chalazion"[Title/Abstract] OR "Ectropion"[Title/Abstract] OR "Entropion"[Title/Abstract] OR "Hordeolum"[Title/Abstract] OR "Meibomian Gland Dysfunction"[Title/Abstract] OR "Trichiasis"[Title/Abstract] OR "Eyelid Disease*"[Title/Abstract] OR "Dacryocystitis"[Title/Abstract] OR "Dry Eye Syndrome*"[Title/Abstract] OR "Lacrimal Duct Obstruction"[Title/Abstract] OR "Lacrimal Apparatus Disease*"[Title/Abstract] OR "Aphakia"[Title/Abstract] OR "Artificial Lens Implant Migration"[Title/Abstract] OR "Cataract"[Title/Abstract] OR "Lens Subluxation"[Title/Abstract] OR "Lens Disease*"[Title/Abstract] OR "Glaucoma"[Title/Abstract] OR "Ocular Hypertension"[Title/Abstract] OR "Ocular Hypotension"[Title/Abstract] OR "Miller Fisher Syndrome"[Title/Abstract] OR "Pathologic Nystagmus"[Title/Abstract] OR "Oculomotor Nerve Disease*"[Title/Abstract] OR "Ophthalmoplegia"[Title/Abstract] OR "Opsoclonus-Myoclonus Syndrome"[Title/Abstract] OR "Strabismus"[Title/Abstract] OR "Tolosa-Hunt Syndrome"[Title/Abstract] OR "Ocular Motility Disorder*"[Title/Abstract] OR "Low Tension Glaucoma"[Title/Abstract] OR "Optic Atrophy"[Title/Abstract] OR "Optic Disk Drusen"[Title/Abstract] OR "Optic Nerve Injur*"[Title/Abstract] OR "Optic Nerve Neoplasm*"[Title/Abstract] OR "Optic Neuritis"[Title/Abstract] OR "Ischemic Optic Neuropathy"[Title/Abstract] OR "Papilledema"[Title/Abstract] OR "Optic Nerve Disease*"[Title/Abstract] OR "Enophthalmos"[Title/Abstract] OR "Exophthalmos"[Title/Abstract] OR "Orbital Cellulitis"[Title/Abstract] OR "Orbital Myositis"[Title/Abstract] OR "Orbital Pseudotumor"[Title/Abstract] OR "Pott Puffy Tumor"[Title/Abstract] OR "Retrobulbar Hemorrhage"[Title/Abstract] OR "Orbital Disease*"[Title/Abstract] OR "Anisocoria"[Title/Abstract] OR "Miosis"[Title/Abstract] OR "Mydriasis"[Title/Abstract] OR "Tonic Pupil"[Title/Abstract] OR "Pupil Disorder*"[Title/Abstract] OR "Aniseikonia"[Title/Abstract] OR "Anisometropia"[Title/Abstract] OR "Astigmatism"[Title/Abstract] OR "Hyperopia"[Title/Abstract] OR "Myopia"[Title/Abstract] OR "Presbyopia"[Title/Abstract] OR "Refractive Error*"[Title/Abstract] OR "Angioid Streaks"[Title/Abstract] OR "Central Serous Chorioretinopathy"[Title/Abstract] OR "Cone Dystrophy"[Title/Abstract] OR "Diabetic Retinopathy"[Title/Abstract] OR "Epiretinal Membrane"[Title/Abstract] OR "Hypertensive Retinopathy"[Title/Abstract] OR "Retinal Artery Occlusion"[Title/Abstract] OR "Retinal Detachment"[Title/Abstract] OR "Retinal Neovascularization"[Title/Abstract] OR "Retinal Perforations"[Title/Abstract] OR "Retinal Telangiectasis"[Title/Abstract] OR "Retinal Vasculitis"[Title/Abstract] OR "Retinal Vein Occlusion"[Title/Abstract] OR "Retinitis"[Title/Abstract] OR "Retinopathy of Prematurity"[Title/Abstract] OR "Proliferative Vitreoretinopathy"[Title/Abstract] OR "Retinal Disease*"[Title/Abstract] OR "Scleritis"[Title/Abstract] OR "Scleral Disease*"[Title/Abstract] OR "Choroid Disease*"[Title/Abstract] OR "Choroid Neoplasms"[Title/Abstract] OR "Choroidal Neovascularization"[Title/Abstract] OR "Choroiditis"[Title/Abstract] OR "Iris Disease*"[Title/Abstract] OR "Exfoliation Syndrome"[Title/Abstract] OR "Iridocyclitis"[Title/Abstract] OR "Iris Neoplasms"[Title/Abstract] OR "Iritis"[Title/Abstract] OR "Uveitis"[Title/Abstract] OR "Uveal Disease*"[Title/Abstract] OR "Amblyopia"[Title/Abstract] OR "Blindness"[Title/Abstract] OR "Color Vision Defects"[Title/Abstract] OR "Diplopia"[Title/Abstract] OR "Night Blindness"[Title/Abstract] OR "Photophobia"[Title/Abstract] OR "Scotoma"[Title/Abstract] OR "Susac Syndrome"[Title/Abstract] OR "Low Vision"[Title/Abstract] OR "Vision Disorder*"[Title/Abstract] OR "Vitreous Detachment"[Title/Abstract])

**Search terms for Web of Science**

((TS=("Large Language Model*" OR "LLM" OR "Chatbot*" OR "ChatGPT" OR "Generative AI"))

AND (TS=("ophthalmolog*" OR "Eye" OR "Asthenopia" OR "Cogan Syndrome" OR "Conjunctival Neoplasms" OR "Conjunctivitis" OR "Pemphigoid" OR "Benign Mucous Membrane" OR "Pinguecula" OR "Pterygium" OR "Xerophthalmia" OR "Conjunctival Disease*" OR "Hereditary Corneal Dystroph*" OR "Corneal Edema" OR "Corneal Endothelial Cell Loss" OR "Corneal Injuries" OR "Corneal Neovascularization" OR "Corneal Opacity" OR "Corneal Wavefront Aberration" OR "Iridocorneal Endothelial Syndrome" OR "Keratitis" OR "Keratoconus" OR "Limbal Stem Cell Deficiency" OR "Trachoma" OR "Corneal Disease*" OR "Aniridia" OR "Anophthalmos" OR "Blepharophimosis" OR "Choroidal Effusion*" OR "Coloboma" OR "Ectopia Lentis" OR "Familial Exudative Vitreoretinopathies" OR "Fraser Syndrome" OR "Hydrophthalmos" OR "Microphthalmos" OR "Persistent Hyperplastic Primary Vitreous" OR "Retinal Dysplasia" OR "Eye Abnormalit*" OR "Aicardi Syndrome" OR "Albinism" OR "Choroideremia" OR "Cone Dystroph*" OR "Cone-Rod Dystroph*" OR "Duane Retraction Syndrome" OR "Familial Exudative Vitreoretinopath*" OR "Graves Ophthalmopathy" OR "Gyrate Atrophy" OR "Leber Congenital Amaurosis" OR "Hereditary Optic Atroph*" OR "Optic Nerve Hypoplasia" OR "Retinal Degeneration" OR "Retinitis Pigmentosa" OR "Retinoblastoma" OR "Stargardt Disease" OR "Walker-Warburg Syndrome" OR "Weill-Marchesani Syndrome" OR "Hereditary Eye Disease*" OR "Choroid Hemorrhage" OR "Hyphema" OR "Retinal Hemorrhage" OR "Vitreous Hemorrhage" OR "Eye Hemorrhage" OR "Corneal Ulcer" OR "Endophthalmitis" OR "Bacterial Eye Infection*" OR "Fungal Eye Infection*" OR "Parasitic Eye Infection*" OR "Viral Eye Infection*" OR "Eye Infection*" OR "Anterior Capsular Rupture" OR "Corneal Injur*" OR "Posterior Capsular Rupture" OR "Eye Injur*" OR "Eye Pain" OR "Lagophthalmos" OR "Eye Manifestation*" OR "Eyelid Neoplasms" OR "Orbital Neoplasms" OR "Paraneoplastic Syndrome" OR "Retinal Neoplasms" OR "Uveal Neoplasms" OR "Eye Neoplasm*" OR "Blepharitis" OR "Blepharoptosis" OR "Blepharospasm" OR "Chalazion" OR "Ectropion" OR "Entropion" OR "Hordeolum" OR "Meibomian Gland Dysfunction" OR "Trichiasis" OR "Eyelid Disease*" OR "Dacryocystitis" OR "Dry Eye Syndrome*" OR "Lacrimal Duct Obstruction" OR "Lacrimal Apparatus Disease*" OR "Aphakia" OR "Artificial Lens Implant Migration" OR "Cataract" OR "Lens Subluxation" OR "Lens Disease*" OR "Glaucoma" OR "Ocular Hypertension" OR "Ocular Hypotension" OR "Miller Fisher Syndrome" OR "Pathologic Nystagmus" OR "Oculomotor Nerve Disease*" OR "Ophthalmoplegia" OR "Opsoclonus-Myoclonus Syndrome" OR "Strabismus" OR "Tolosa-Hunt Syndrome" OR "Ocular Motility Disorder*" OR "Low Tension Glaucoma" OR "Optic Atrophy" OR "Optic Disk Drusen" OR "Optic Nerve Injur*" OR "Optic Nerve Neoplasm*" OR "Optic Neuritis" OR "Ischemic Optic Neuropathy" OR "Papilledema" OR "Optic Nerve Disease*" OR "Enophthalmos" OR "Exophthalmos" OR "Orbital Cellulitis" OR "Orbital Myositis" OR "Orbital Pseudotumor" OR "Pott Puffy Tumor" OR "Retrobulbar Hemorrhage" OR "Orbital Disease*" OR "Anisocoria" OR "Miosis" OR "Mydriasis" OR "Tonic Pupil" OR "Pupil Disorder*" OR "Aniseikonia" OR "Anisometropia" OR "Astigmatism" OR "Hyperopia" OR "Myopia" OR "Presbyopia" OR "Refractive Error*" OR "Angioid Streaks" OR "Central Serous Chorioretinopathy" OR "Cone Dystrophy" OR "Diabetic Retinopathy" OR "Epiretinal Membrane" OR "Hypertensive Retinopathy" OR "Retinal Artery Occlusion" OR "Retinal Detachment" OR "Retinal Neovascularization" OR "Retinal Perforations" OR "Retinal Telangiectasis" OR "Retinal Vasculitis" OR "Retinal Vein Occlusion" OR "Retinitis" OR "Retinopathy of Prematurity" OR "Proliferative Vitreoretinopathy" OR "Retinal Disease*" OR "Scleritis" OR "Scleral Disease*" OR "Choroid Disease*" OR "Choroid Neoplasms" OR "Choroidal Neovascularization" OR "Choroiditis" OR "Iris Disease*" OR "Exfoliation Syndrome" OR "Iridocyclitis" OR "Iris Neoplasms" OR "Iritis" OR "Uveitis" OR "Uveal Disease*" OR "Amblyopia" OR "Blindness" OR "Color Vision Defects" OR "Diplopia" OR "Night Blindness" OR "Photophobia" OR "Scotoma" OR "Susac Syndrome" OR "Low Vision" OR "Vision Disorder*" OR "Vitreous Detachment")))

**Search terms for Embase**

('large language model*':ti,ab OR 'LLM':ti,ab OR 'chatbot*':ti,ab OR 'ChatGPT':ti,ab OR 'generative AI':ti,ab)

AND ('ophthalmolog*':ti,ab OR 'eye':ti,ab OR "Asthenopia":ti,ab OR "Cogan Syndrome":ti,ab OR "Conjunctival Neoplasms":ti,ab OR "Conjunctivitis":ti,ab OR "Pemphigoid":ti,ab OR "Benign Mucous Membrane":ti,ab OR "Pinguecula":ti,ab OR "Pterygium":ti,ab OR "Xerophthalmia":ti,ab OR "Conjunctival Disease*":ti,ab OR "Hereditary Corneal Dystroph*":ti,ab OR "Corneal Edema":ti,ab OR "Corneal Endothelial Cell Loss":ti,ab OR "Corneal Injuries":ti,ab OR "Corneal Neovascularization":ti,ab OR "Corneal Opacity":ti,ab OR "Corneal Wavefront Aberration":ti,ab OR "Iridocorneal Endothelial Syndrome":ti,ab OR "Keratitis":ti,ab OR "Keratoconus":ti,ab OR "Limbal Stem Cell Deficiency":ti,ab OR "Trachoma":ti,ab OR "Corneal Disease*":ti,ab OR "Aniridia":ti,ab OR "Anophthalmos":ti,ab OR "Blepharophimosis":ti,ab OR "Choroidal Effusion*":ti,ab OR "Coloboma":ti,ab OR "Ectopia Lentis":ti,ab OR "Familial Exudative Vitreoretinopathies":ti,ab OR "Fraser Syndrome":ti,ab OR "Hydrophthalmos":ti,ab OR "Microphthalmos":ti,ab OR "Persistent Hyperplastic Primary Vitreous":ti,ab OR "Retinal Dysplasia":ti,ab OR "Eye Abnormalit*":ti,ab OR "Aicardi Syndrome":ti,ab OR "Albinism":ti,ab OR "Choroideremia":ti,ab OR "Cone Dystroph*":ti,ab OR "Cone-Rod Dystroph*":ti,ab OR "Duane Retraction Syndrome":ti,ab OR "Familial Exudative Vitreoretinopath*":ti,ab OR "Graves Ophthalmopathy":ti,ab OR "Gyrate Atrophy":ti,ab OR "Leber Congenital Amaurosis":ti,ab OR "Hereditary Optic Atroph*":ti,ab OR "Optic Nerve Hypoplasia":ti,ab OR "Retinal Degeneration":ti,ab OR "Retinitis Pigmentosa":ti,ab OR "Retinoblastoma":ti,ab OR "Stargardt Disease":ti,ab OR "Walker-Warburg Syndrome":ti,ab OR "Weill-Marchesani Syndrome":ti,ab OR "Hereditary Eye Disease*":ti,ab OR "Choroid Hemorrhage":ti,ab OR "Hyphema":ti,ab OR "Retinal Hemorrhage":ti,ab OR "Vitreous Hemorrhage":ti,ab OR "Eye Hemorrhage":ti,ab OR "Corneal Ulcer":ti,ab OR "Endophthalmitis":ti,ab OR "Bacterial Eye Infection*":ti,ab OR "Fungal Eye Infection*":ti,ab OR "Parasitic Eye Infection*":ti,ab OR "Viral Eye Infection*":ti,ab OR "Eye Infection*":ti,ab OR "Anterior Capsular Rupture":ti,ab OR "Corneal Injur*":ti,ab OR "Posterior Capsular Rupture":ti,ab OR "Eye Injur*":ti,ab OR "Eye Pain":ti,ab OR "Lagophthalmos":ti,ab OR "Eye Manifestation*":ti,ab OR "Eyelid Neoplasms":ti,ab OR "Orbital Neoplasms":ti,ab OR "Paraneoplastic Syndrome":ti,ab OR "Retinal Neoplasms":ti,ab OR "Uveal Neoplasms":ti,ab OR "Eye Neoplasm*":ti,ab OR "Blepharitis":ti,ab OR "Blepharoptosis":ti,ab OR "Blepharospasm":ti,ab OR "Chalazion":ti,ab OR "Ectropion":ti,ab OR "Entropion":ti,ab OR "Hordeolum":ti,ab OR "Meibomian Gland Dysfunction":ti,ab OR "Trichiasis":ti,ab OR "Eyelid Disease*":ti,ab OR "Dacryocystitis":ti,ab OR "Dry Eye Syndrome*":ti,ab OR "Lacrimal Duct Obstruction":ti,ab OR "Lacrimal Apparatus Disease*":ti,ab OR "Aphakia":ti,ab OR "Artificial Lens Implant Migration":ti,ab OR "Cataract":ti,ab OR "Lens Subluxation":ti,ab OR "Lens Disease*":ti,ab OR "Glaucoma":ti,ab OR "Ocular Hypertension":ti,ab OR "Ocular Hypotension":ti,ab OR "Miller Fisher Syndrome":ti,ab OR "Pathologic Nystagmus":ti,ab OR "Oculomotor Nerve Disease*":ti,ab OR "Ophthalmoplegia":ti,ab OR "Opsoclonus-Myoclonus Syndrome":ti,ab OR "Strabismus":ti,ab OR "Tolosa-Hunt Syndrome":ti,ab OR "Ocular Motility Disorder*":ti,ab OR "Low Tension Glaucoma":ti,ab OR "Optic Atrophy":ti,ab OR "Optic Disk Drusen":ti,ab OR "Optic Nerve Injur*":ti,ab OR "Optic Nerve Neoplasm*":ti,ab OR "Optic Neuritis":ti,ab OR "Ischemic Optic Neuropathy":ti,ab OR "Papilledema":ti,ab OR "Optic Nerve Disease*":ti,ab OR "Enophthalmos":ti,ab OR "Exophthalmos":ti,ab OR "Orbital Cellulitis":ti,ab OR "Orbital Myositis":ti,ab OR "Orbital Pseudotumor":ti,ab OR "Pott Puffy Tumor":ti,ab OR "Retrobulbar Hemorrhage":ti,ab OR "Orbital Disease*":ti,ab OR "Anisocoria":ti,ab OR "Miosis":ti,ab OR "Mydriasis":ti,ab OR "Tonic Pupil":ti,ab OR "Pupil Disorder*":ti,ab OR "Aniseikonia":ti,ab OR "Anisometropia":ti,ab OR "Astigmatism":ti,ab OR "Hyperopia":ti,ab OR "Myopia":ti,ab OR "Presbyopia":ti,ab OR "Refractive Error*":ti,ab OR "Angioid Streaks":ti,ab OR "Central Serous Chorioretinopathy":ti,ab OR "Cone Dystrophy":ti,ab OR "Diabetic Retinopathy":ti,ab OR "Epiretinal Membrane":ti,ab OR "Hypertensive Retinopathy":ti,ab OR "Retinal Artery Occlusion":ti,ab OR "Retinal Detachment":ti,ab OR "Retinal Neovascularization":ti,ab OR "Retinal Perforations":ti,ab OR "Retinal Telangiectasis":ti,ab OR "Retinal Vasculitis":ti,ab OR "Retinal Vein Occlusion":ti,ab OR "Retinitis":ti,ab OR "Retinopathy of Prematurity":ti,ab OR "Proliferative Vitreoretinopathy":ti,ab OR "Retinal Disease*":ti,ab OR "Scleritis":ti,ab OR "Scleral Disease*":ti,ab OR "Choroid Disease*":ti,ab OR "Choroid Neoplasms":ti,ab OR "Choroidal Neovascularization":ti,ab OR "Choroiditis":ti,ab OR "Iris Disease*":ti,ab OR "Exfoliation Syndrome":ti,ab OR "Iridocyclitis":ti,ab OR "Iris Neoplasms":ti,ab OR "Iritis":ti,ab OR "Uveitis":ti,ab OR "Uveal Disease*":ti,ab OR "Amblyopia":ti,ab OR "Blindness":ti,ab OR "Color Vision Defects":ti,ab OR "Diplopia":ti,ab OR "Night Blindness":ti,ab OR "Photophobia":ti,ab OR "Scotoma":ti,ab OR "Susac Syndrome":ti,ab OR "Low Vision":ti,ab OR "Vision Disorder*":ti,ab OR "Vitreous Detachment":ti,ab)

**Search terms for IEEEXplore**

("Large Language Model" OR "LLM" OR "Chatbot" OR "ChatGPT" OR "Generative AI")

AND ("ophthalmology" OR "eye" OR "Asthenopia" OR "Cogan Syndrome" OR "Conjunctival Neoplasms" OR "Conjunctivitis" OR "Pemphigoid" OR "Benign Mucous Membrane" OR "Pinguecula" OR "Pterygium" OR "Xerophthalmia" OR "Conjunctival Diseases" OR "Hereditary Corneal Dystrophies" OR "Corneal Edema" OR "Corneal Endothelial Cell Loss" OR "Corneal Injuries" OR "Corneal Neovascularization" OR "Corneal Opacity" OR "Corneal Wavefront Aberration" OR "Iridocorneal Endothelial Syndrome" OR "Keratitis" OR "Keratoconus" OR "Limbal Stem Cell Deficiency" OR "Trachoma" OR "Corneal Diseases" OR "Aniridia" OR "Anophthalmos" OR "Blepharophimosis" OR "Choroidal Effusions" OR "Coloboma" OR "Ectopia Lentis" OR "Familial Exudative Vitreoretinopathies" OR "Fraser Syndrome" OR "Hydrophthalmos" OR "Microphthalmos" OR "Persistent Hyperplastic Primary Vitreous" OR "Retinal Dysplasia" OR "Eye Abnormalities" OR "Aicardi Syndrome" OR "Albinism" OR "Choroideremia" OR "Cone Dystrophy" OR "Cone-Rod Dystrophies" OR "Duane Retraction Syndrome" OR "Graves Ophthalmopathy" OR "Gyrate Atrophy" OR "Leber Congenital Amaurosis" OR "Hereditary Optic Atrophies" OR "Optic Nerve Hypoplasia" OR "Retinal Degeneration" OR "Retinitis Pigmentosa" OR "Retinoblastoma" OR "Stargardt Disease" OR "Walker-Warburg Syndrome" OR "Weill-Marchesani Syndrome" OR "Hereditary Eye Diseases" OR "Choroid Hemorrhage" OR "Hyphema" OR "Retinal Hemorrhage" OR "Vitreous Hemorrhage" OR "Eye Hemorrhage" OR "Corneal Ulcer" OR "Endophthalmitis" OR "Bacterial Eye Infections" OR "Fungal Eye Infections" OR "Parasitic Eye Infections" OR "Eye Infections" OR "Anterior Capsular Rupture" OR "Posterior Capsular Rupture" OR "Eye Injuries" OR "Eye Pain" OR "Lagophthalmos" OR "Eye Manifestations" OR "Eyelid Neoplasms" OR "Orbital Neoplasms" OR "Paraneoplastic Syndrome" OR "Retinal Neoplasms" OR "Uveal Neoplasms" OR "Eye Neoplasms" OR "Blepharitis" OR "Blepharoptosis" OR "Blepharospasm" OR "Chalazion" OR "Ectropion" OR "Entropion" OR "Hordeolum" OR "Meibomian Gland Dysfunction" OR "Trichiasis" OR "Eyelid Diseases" OR "Dacryocystitis" OR "Dry Eye Syndromes" OR "Lacrimal Duct Obstruction" OR "Lacrimal Apparatus Diseases" OR "Aphakia" OR "Artificial Lens Implant Migration" OR "Cataract" OR "Lens Subluxation" OR "Lens Diseases" OR "Glaucoma" OR "Ocular Hypertension" OR "Ocular Hypotension" OR "Miller Fisher Syndrome" OR "Pathologic Nystagmus" OR "Oculomotor Nerve Diseases" OR "Ophthalmoplegia" OR "Opsoclonus-Myoclonus Syndrome" OR "Strabismus" OR "Tolosa-Hunt Syndrome" OR "Ocular Motility Disorders" OR "Low Tension Glaucoma" OR "Optic Atrophy" OR "Optic Disk Drusen" OR "Optic Nerve Injuries" OR "Optic Nerve Neoplasms" OR "Optic Neuritis" OR "Ischemic Optic Neuropathy" OR "Papilledema" OR "Optic Nerve Diseases" OR "Enophthalmos" OR "Exophthalmos" OR "Orbital Cellulitis" OR "Orbital Myositis" OR "Orbital Pseudotumor" OR "Pott Puffy Tumor" OR "Retrobulbar Hemorrhage" OR "Orbital Diseases" OR "Anisocoria" OR "Miosis" OR "Mydriasis" OR "Tonic Pupil" OR "Pupil Disorders" OR "Aniseikonia" OR "Anisometropia" OR "Astigmatism" OR "Hyperopia" OR "Myopia" OR "Presbyopia" OR "Refractive Errors" OR "Angioid Streaks" OR "Central Serous Chorioretinopathy" OR "Diabetic Retinopathy" OR "Epiretinal Membrane" OR "Hypertensive Retinopathy" OR "Retinal Artery Occlusion" OR "Retinal Detachment" OR "Retinal Neovascularization" OR "Retinal Perforations" OR "Retinal Telangiectasis" OR "Retinal Vasculitis" OR "Retinal Vein Occlusion" OR "Retinitis" OR "Retinopathy of Prematurity" OR "Proliferative Vitreoretinopathy" OR "Retinal Diseases" OR "Scleritis" OR "Scleral Diseases" OR "Choroid Diseases" OR "Choroid Neoplasms" OR "Choroidal Neovascularization" OR "Choroiditis" OR "Iris Diseases" OR "Exfoliation Syndrome" OR "Iridocyclitis" OR "Iris Neoplasms" OR "Iritis" OR "Uveitis" OR "Uveal Diseases" OR "Amblyopia" OR "Blindness" OR "Color Vision Defects" OR "Diplopia" OR "Night Blindness" OR "Photophobia" OR "Scotoma" OR "Susac Syndrome" OR "Low Vision" OR "Vision Disorders" OR "Vitreous Detachment")

**Table S1. Detailed definitions for the 9 “Medical Task” labels.**

| Task label | Detailed definition | Typical example |
| --- | --- | --- |
| Medical query | Asking questions from patients, students or physicians that seek factual ophthalmic information (symptoms, treatments, prognosis, lifestyle advice, etc.) | “Can blepharitis cause blurry vision at night?” |
| Standardized examination | Finishing exam questions extracted from validated test banks or certification quizzes | American Board of Ophthalmology multiple-choice questions. |
| Diagnosis making | Determining final diagnoses or ranking differential diagnoses for ophthalmic cases | Fundus photo + history → “What is the most likely diagnosis?” |
| Ophthalmic education | Writing new or modifying existing PEMs for clear understanding. This category differs from "Medical query" in that it emphasizes assessing the ​readability and usability​ of the generated PEMs | “Explain the pathogenesis of glaucoma to patients in highly readable text.” |
| Clinical decision making | Advice on investigations, treatment plans or follow-up schedules for specific patient scenario | “Recommend next-step management for a 65-year-old with newly diagnosed neovascular AMD.” |
| Medical text generation | Drafting operative notes, discharge summaries or other clinical documents | “Construct discharge summaries for this post-cataract surgery patient.” |
| Research assisting | Literature search, abstract summarization, code generation or data analysis for ophthalmic research projects. | “Generate scientific abstracts with 10 references for neuro-ophthalmology.” |
| Patient triaging | Classifying urgency or directing patients to the appropriate service level based on symptoms or images. | “Determine ‘emergency department vs routine clinic’ referral for this patient with acute ophthalmological symptoms” |
| Disease prediction | Output is a future risk probability (e.g., vision loss, disease progression) derived from textual or multimodal data. | Predict 2-year conversion from dry to wet AMD. |

Abbreviations: AMD, age-related macular degeneration; PEMs, patient education materials

**Table S2. Study‑level input data and effect‑size calculations for the diagnosis‑making task meta‑analysis.**

| Study No. | Reference | Model | k correct | N total | Subspeciality | Modality |
| --- | --- | --- | --- | --- | --- | --- |
| 1 | Delsoz et al. [1] | ChatGPT-3.5 | 8 | 11 | Glaucoma | Text |
| 2 | Delsoz et al. [2] | ChatGPT-3.5 | 12 | 20 | External Disease & Cornea | Text |
| 2 | Delsoz et al. [2] | ChatGPT-4 | 17 | 20 | External Disease & Cornea | Text |
| 3 | Rojas-Carabali et al. [3] | ChatGPT-3.5 | 15 | 25 | Uveitis & Ocular Inflammation | Text |
| 3 | Rojas-Carabali et al. [3] | ChatGPT-3.5 | 15 | 25 | Uveitis & Ocular Inflammation | Text |
| 4 | Madadi et al. [4] | ChatGPT-3.5 | 13 | 22 | Neuro-Ophthalmology | Text |
| 4 | Madadi et al. [4] | ChatGPT-3.5 | 18 | 22 | Neuro-Ophthalmology | Text |
| 5 | Shemer et al. [5] | ChatGPT-3.5 | 43 | 63 | Comprehensive Ophthalmology | Text |
| 6 | Ghalibafan et al. [6] | ChatGPT-4 | 35 | 256 | Retina & Vitreous | Vision-Text |
| 7 | Rojas-Carabali et al. [7] | ChatGPT-3.5 | 4 | 6 | Uveitis & Ocular Inflammation | Text |
| 7 | Rojas-Carabali et al. [7] | ChatGPT-4 | 4 | 6 | Uveitis & Ocular Inflammation | Text |
| 7 | Rojas-Carabali et al. [7] | Glass 1.0 | 2 | 6 | Uveitis & Ocular Inflammation | Text |
| 8 | Milad et al. [8] | ChatGPT-4 | 203 | 422 | Comprehensive Ophthalmology | Text |
| 9 | Shukla et al. [9] | ChatGPT-3.5 | 4 | 10 | Neuro-Ophthalmology | Text |
| 9 | Shukla et al. [9] | Bing Copilot | 4 | 10 | Neuro-Ophthalmology | Text |
| 9 | Shukla et al. [9] | Gemini | 4 | 10 | Neuro-Ophthalmology | Text |
| 10 | Ming et al. [10] | ChatGPT-3.5 | 41 | 104 | Comprehensive Ophthalmology | Text |
| 10 | Ming et al. [10] | ChatGPT-4 | 62 | 104 | Comprehensive Ophthalmology | Text |
| 11 | Hu et al. [11] | ChatGPT-4 | 9 | 10 | Comprehensive Ophthalmology | Text |
| 12 | Zandi et al. [12] | ChatGPT-4 | 43 | 80 | Comprehensive Ophthalmology | Text |
| 12 | Zandi et al. [12] | Bard | 35 | 80 | Comprehensive Ophthalmology | Text |
| 13 | Mandalos et al. [13] | ChatGPT-3.5 | 7 | 9 | Comprehensive Ophthalmology | Text |
| 13 | Mandalos et al. [13] | Bing Copilot | 6 | 9 | Comprehensive Ophthalmology | Text |
| 13 | Mandalos et al. [13] | Gemini | 4 | 9 | Comprehensive Ophthalmology | Text |
| 14 | Liu et al. [14] | ChatGPT-3.5 | 1019 | 1226 | Retina & Vitreous | Text |
| 15 | Sorin et al. [15] | ChatGPT-4 | 27 | 40 | Comprehensive Ophthalmology | Vision-Text |
| 16 | Mihalache et al. [16] | ChatGPT-4 | 35 | 69 | Retina & Vitreous | Vision-Text |
| 17 | Zheng et al. [17] | MOPH | 146 | 180 | Comprehensive Ophthalmology | Text |

**
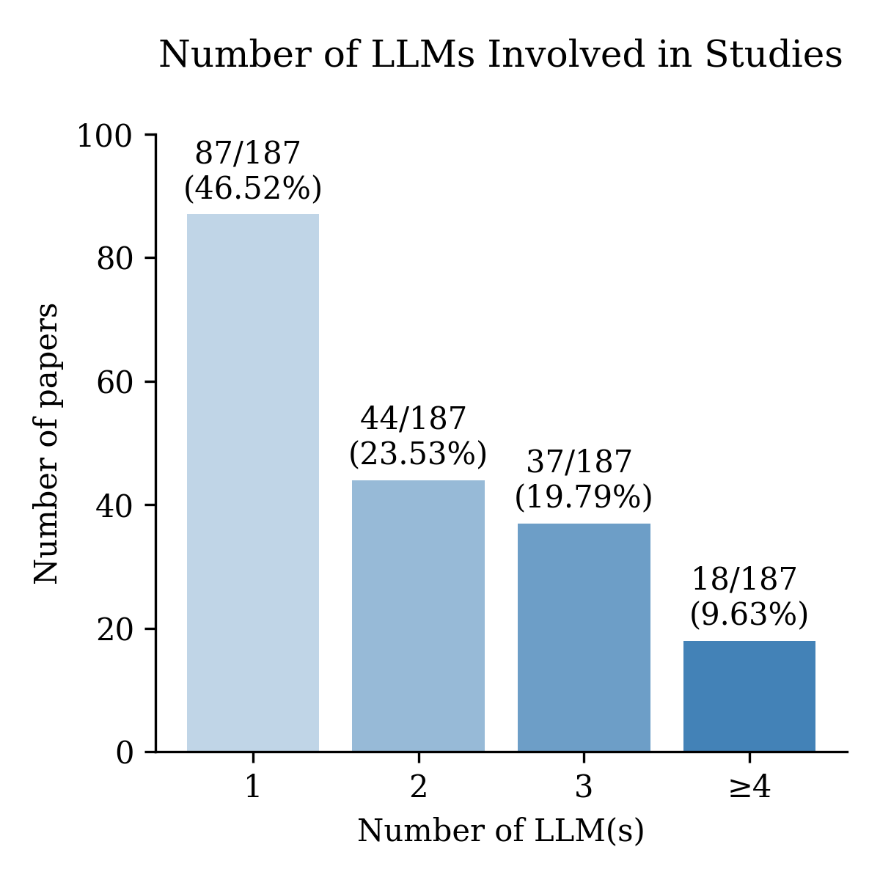
**

**Figure S1. Number of LLMs evaluated per included study.**


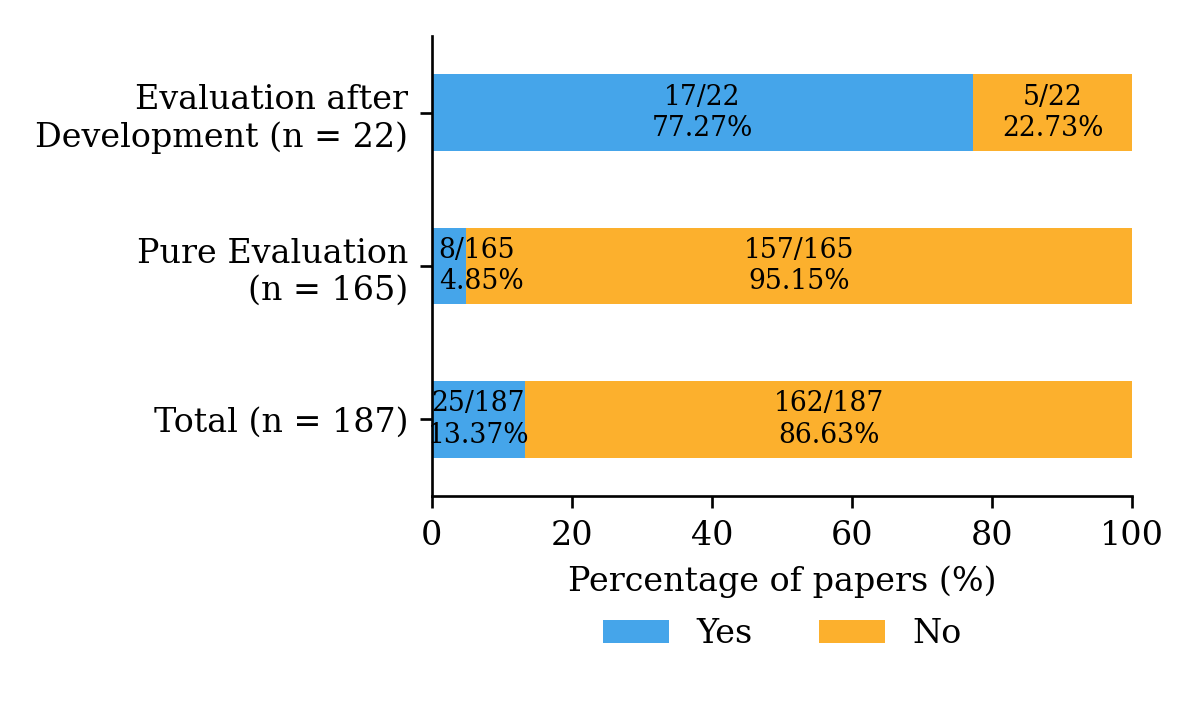


**Figure S2. Proportion of studies evaluating open-source LLMs.**


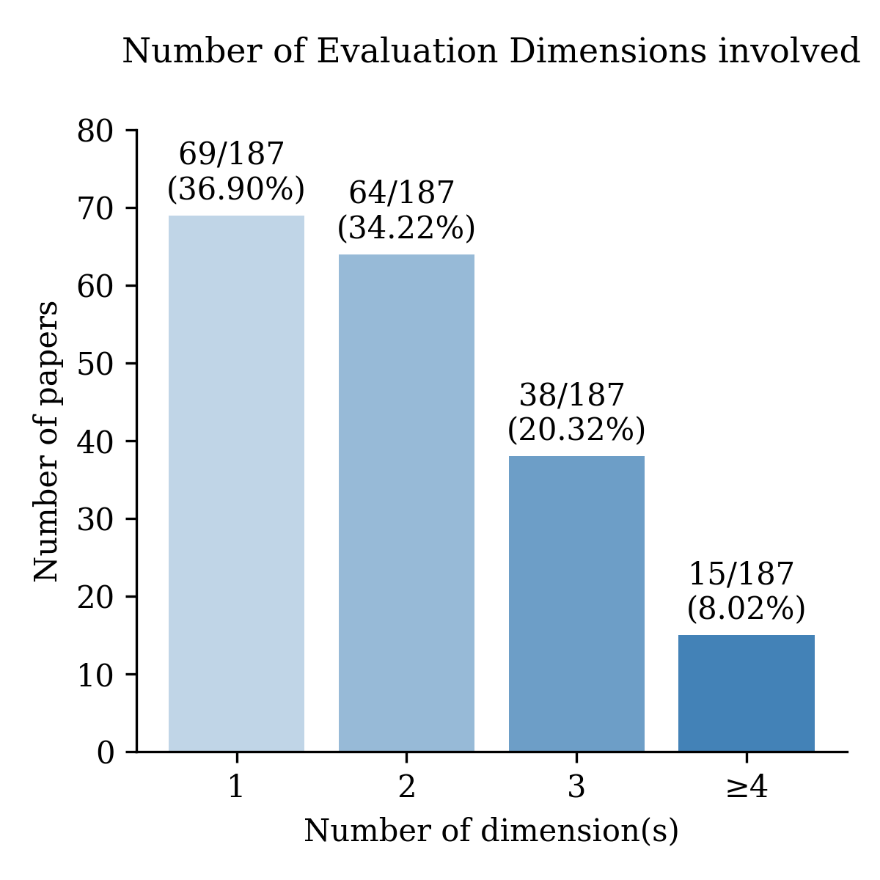


**Figure S3. Number of evaluation dimensions per included study.**


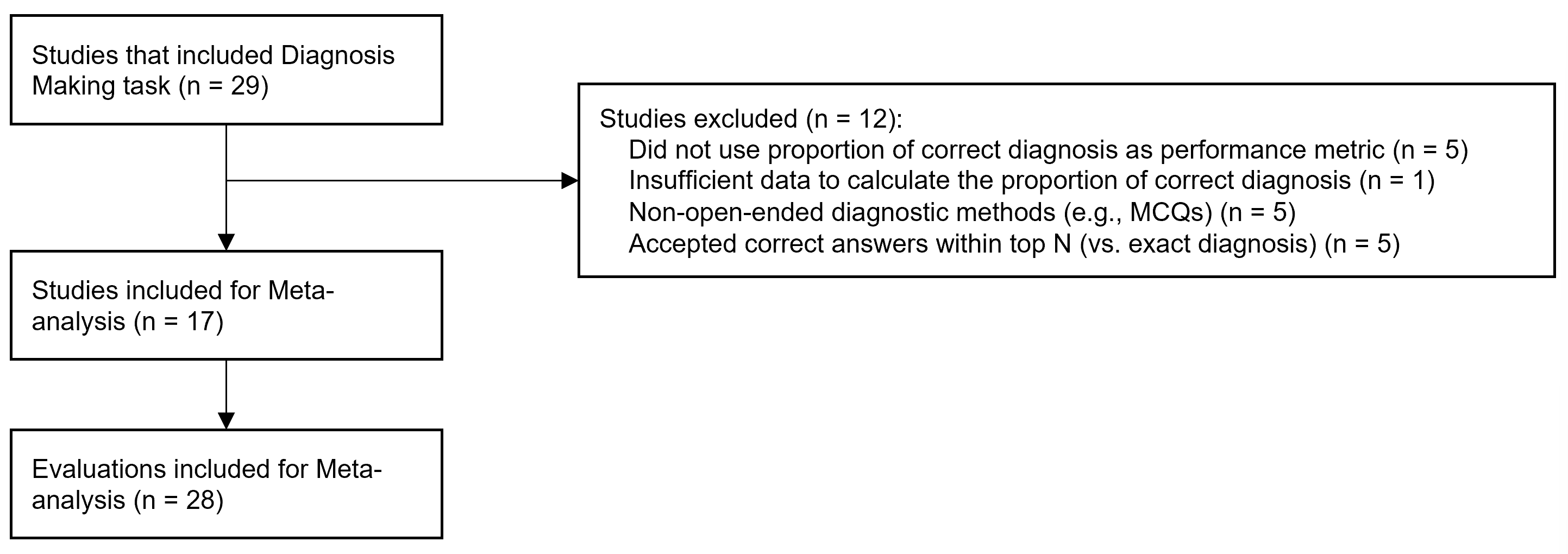


**Figure S4. Study selection flow diagram for the diagnosis task meta‑analysis.**


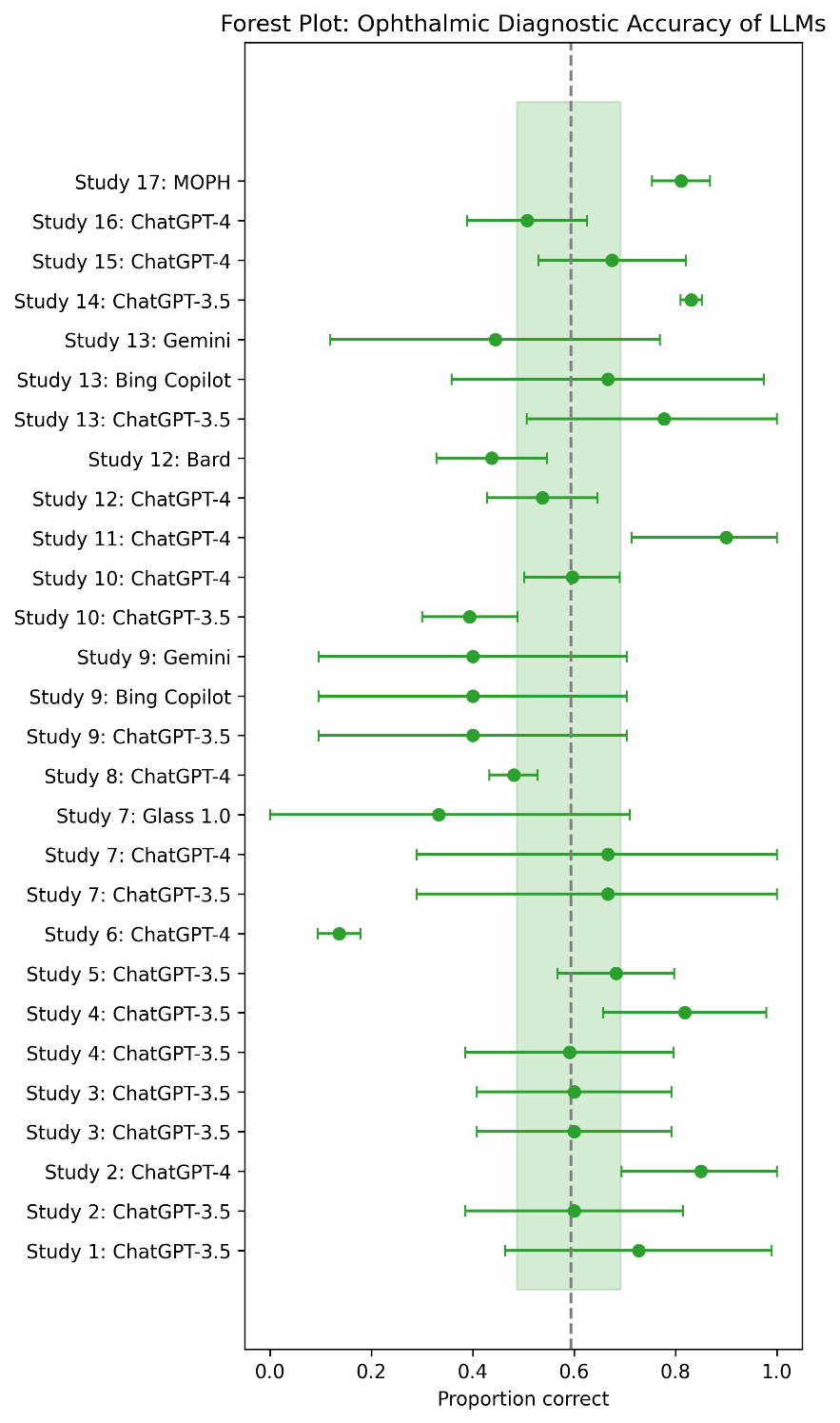


**Figure S5. Forest plot of pooled diagnostic accuracy for diagnosis-making tasks**

**Reference**

[1] Delsoz, Mohammad, et al. "The use of ChatGPT to assist in diagnosing glaucoma based on clinical case reports." *Ophthalmology and therapy* 12.6 (2023): 3121-3132.

[2] Delsoz, Mohammad, et al. "Performance of ChatGPT in diagnosis of corneal eye diseases." Cornea 43.5 (2024): 664-670.

[3] Rojas-Carabali, William, et al. "Evaluating the diagnostic accuracy and management recommendations of ChatGPT in uveitis." *Ocular immunology and inflammation* 32.8 (2024): 1526-1531.

[4] Madadi, Yeganeh, et al. "ChatGPT assisting diagnosis of neuro-ophthalmology diseases based on case reports." *Journal of Neuro-Ophthalmology* (2022): 10-1097.

[5] Shemer, Asaf, et al. "Diagnostic capabilities of ChatGPT in ophthalmology." *Graefe's Archive for Clinical and Experimental Ophthalmology* 262.7 (2024): 2345-2352.

[6] Ghalibafan, Seyyedehfatemeh, et al. "Applications of multimodal generative artificial intelligence in a real-world retina clinic setting." *Retina* 44.10 (2024): 1732-1740.

[7] Rojas-Carabali, William, et al. "Chatbots vs. human experts: evaluating diagnostic performance of chatbots in uveitis and the perspectives on AI adoption in ophthalmology." Ocular Immunology and Inflammation 32.8 (2024): 1591-1598.

[8] Milad, Daniel, et al. "Assessing the medical reasoning skills of GPT-4 in complex ophthalmology cases." British Journal of Ophthalmology 108.10 (2024): 1398-1405.

[9] Shukla, Ruchi, et al. "The comparison of ChatGPT 3.5, Microsoft Bing, and Google Gemini for diagnosing cases of neuro-ophthalmology." Cureus 16.4 (2024).

[11] Ming, Shuai, et al. "Performance of ChatGPT in ophthalmic registration and clinical diagnosis: cross-sectional study." Journal of Medical Internet Research 26 (2024): e60226.

[11] Hu, Xiaoyan, et al. "What can GPT-4 do for diagnosing rare eye diseases? A pilot study." Ophthalmology and Therapy 12.6 (2023): 3395-3402.

[12] Zandi, Roya, et al. "Exploring diagnostic precision and triage proficiency: a comparative study of GPT-4 and Bard in addressing common ophthalmic complaints." Bioengineering 11.2 (2024): 120.

[13] Mandalos, Achilleas, and Dimitrios Tsouris. "Artificial versus human intelligence in the diagnostic approach of ophthalmic case scenarios: a qualitative evaluation of performance and consistency." Cureus 16.6 (2024).

[14] Liu, Xiaocong, et al. "Transforming retinal vascular disease classification: a comprehensive analysis of chatgpt’s performance and inference abilities on non-english clinical environment." medRxiv (2023): 2023-06.

[15] Sorin, Vera, et al. "GPT-4 multimodal analysis on ophthalmology clinical cases including text and images." medRxiv (2023): 2023-11.

[16] Mihalache, Andrew, et al. "Interpretation of clinical retinal images using an artificial intelligence Chatbot." Ophthalmology Science 4.6 (2024): 100556.

[17] Zheng, Ce, et al. "Development and evaluation of a large language model of ophthalmology in Chinese." British Journal of Ophthalmology 108.10 (2024): 1390-1397.

[18] Gill, Gurnoor S., Jacob Blair, and Steven Litinsky. "Evaluating the performance of ChatGPT 3.5 and 4.0 on StatPearls oculoplastic surgery text-and image-based exam questions." *Cureus* 16.11 (2024).

[19] Xu, P. et al. "Unveiling the Clinical Incapabilities: A Benchmarking Study of Gpt-4v(Ision) for Ophthalmic Multimodal Image Analysis." *British Journal of Ophthalmology*, 2024, Publisher, doi:10.1136/bjo-2023-325054.
